# Metal-induced delayed type hypersensitivity responses potentiate particle induced osteolysis in a sex and age dependent manner

**DOI:** 10.1371/journal.pone.0251885

**Published:** 2021-05-18

**Authors:** Lauryn Samelko, Marco Caicedo, Kyron McAllister, Joshua Jacobs, Nadim James Hallab

**Affiliations:** Department of Orthopedic Surgery, Rush University Medical Center, Chicago, IL, United States of America; University of Oulu, FINLAND

## Abstract

It is widely recognized that innate macrophage immune reactions to implant debris are central to the inflammatory responses that drive biologic implant failure over the long term. Less common, adaptive lymphocyte immune reactions to implant debris, such as delayed type hypersensitivity (DTH), can also affect implant performance. It is unknown which key patient factors, if any, mediate these adaptive immune responses that potentiate particle/macrophage mediated osteolysis. The objective of this investigation was to determine to what degree known adaptive immune responses to metal implant debris can affect particle-induced osteolysis (PIO); and if this pathomechanism is dependent on: 1) innate immune danger signaling, i.e., NLRP3 inflammasome activity, 2) sex, and/or 3) age. We used an established murine calvaria model of PIO using male and female wild-type C57BL/6 vs. Caspase-1 deficient mice as well as young (12–16 weeks old) vs. aged (18–24 months old) female and male C57BL/6 mice. After induction of metal-DTH, and Cobalt-alloy particle (ASTM F-75, 0.4um median diameter) calvaria challenge, bone resorption was assessed using quantitative micro-computed tomography (micro-CT) analysis and immune responses were assessed by measuring paw inflammation, lymphocyte transformation test (LTT) reactivity and adaptive immune cytokines IFN-gamma and IL-17 (ELISA). Younger aged C57BL/6 female mice exhibited the highest rate and severity of metal sensitivity lymphocyte responses that also translated into higher PIO compared to any other experimental group. The absence of inflammasome/caspase-1 activity significantly suppressed DTH metal-reactivity and osteolysis in both male and female Caspase-1 deficient mice. These murine model results indicate that young female mice are more predisposed to metal-DTH augmented inflammatory responses to wear debris, which is highly influenced by active NLRP3 inflammasome/caspase-1 danger signaling. If these results are clinically meaningful for orthopedic patients, then younger female individuals should be appropriately assessed and followed for DTH derived peri-implant complications.

## Introduction

The prevalence of total joint arthroplasties (TJAs) within the population is increasing as the number of TJAs performed yearly in the U.S. is expected to rise to 4 million per year by 2030 [1]. Concomitantly, the average age of a TJA individual has been decreasing, while TJA complications associated with younger patients have increased [1]. Periprosthetic osteolysis-associated aseptic loosening remains a major complication that accounts for at least 50% of all total hip arthroplasty (THA) and 44% of total knee arthroplasty (TKA) revisions [2–5].

The time from implantation to aseptic loosening (AL) can drastically differ between individuals with the same implant and the same amount of implant debris. Implant debris produced by prosthetic wear and corrosion are the primary factors that trigger elements of the immune system that lead to bone resorption and eventually implant loosening [6, 7]. Wear particles are recognized and phagocytosed by peri-implant tissue macrophages, consequently triggering a cascade of pro-inflammatory immune system responses by inducing the release of cytokine/chemokine mediators that activate osteoclasts at the bone-implant interface [8]. Recent evidence indicates that some patients have different patterns of innate immune cytokine gene expression to pro-inflammatory stimuli and may be more susceptible to implant debris induced osteolysis [9]. Typically, histologic evidence from retrieved periprosthetic tissues from patients with loosening reveals macrophage inflammatory biological reactions to implant wear particles; with concomitant expression of inflammatory factors such as IL-1β, TNF-α and IL-6 [10, 11]. Of particular concern is IL-1β, a known potent cytokine that can drive cartilage and bone destruction by inducing production of matrix metalloproteinases and degradative products as well by stimulating osteoclast differentiation and augmenting bone resorption [12]. The secretion of mature IL-1β is dependent on innate immune danger signaling, e.g., NLRP3 inflammasome activation of caspase-1 [13]. NLRP3 inflammasome is a critical component of the innate immune system and can be activated by a diverse number of cellular irritants and endogenous damage signals such as ionic flux, reactive oxygen species (ROS), mitochondrial and lysosomal damage [14–16]. Exogenous danger signals such as silica, aluminum salt crystals and implant wear debris also result in assembly and activation of the NLRP3 inflammasome [13, 17–24]. It has been shown both in vitro and in vivo, that implant wear debris provokes inflammatory cell influx and bone resorption via this danger dedicated sensing/activation mechanism of innate immune cells, where the NLRP3 inflammasome promotes IL-1β secretion in a highly dose-dependent manner [21–23, 25]. Blocking the inflammasome pathway at various checkpoints has been shown to not affect cell metabolism but does shut down the induction of implant wear debris induced inflammation [22–24]. Thus, the inflammatory host response by macrophages of the innate immune system to implant wear particles are a leading pathomechanism driving inflammation and bone loss around implant-tissue interfaces.

When present at abnormally high levels, some types of metal particles can cause metal toxicity-like responses and can also induce adaptive immune responses such as a delayed-type hypersensitivity reaction (DTH), that have been identified systemically and have been described histologically as aseptic lymphocyte dominated vasculitis associated lesions (ALVAL) [26–29]. Although more common to specific types of implants that release metal implant debris (e.g., metal-on-metal (MoM) total hip arthroplasty), adaptive immune responses to implant debris have also been reported in total knee arthroplasty (TKA) and non-MoM implants [29, 30]. Accumulating evidence continues to demonstrate a strong positive correlation between metal exposure, metal-DTH responses and periprosthetic performance [31–42]. This correlation is a result of past cohort studies reporting that the incidence of metal sensitivity among patients with well-functioning implants is approximately 20%; roughly twice as high as that of the general population [31, 33, 35, 36, 39, 40, 43–45]. This rate dramatically increases to 50–60% in patients with a painful or poorly functioning implant and has been shown to be as high as 100% in smaller group studies of revised metal-on-metal (MoM) THAs [31, 33, 35, 36, 39, 40, 43–45]. Additionally, it has been found that women have a 29% higher risk of short-term THA failure [46] and a higher prevalence and severity of DTH lymphocyte reactions to wear debris compared to men [47]. Thus, it has been suggested that metal-activated lymphocytes, of the adaptive immune system, which release potent pro-inflammatory cytokines and bone resorbing mediators may contribute to AL and individual-specific predisposition toward implant failure [48–51]. This is further demonstrated by several studies reporting an association between orthopedic implants and metal DTH reactions, which resolve after implant removal or revision to a more so-called hypoallergenic implant or with the use of immunosuppressive drugs [49, 52–56]. However, it remains unclear to what extent adaptive immune responses can aggravate wear debris induced innate immune responses, and if a pre-existing conditions of metal sensitivity prior to a TJA increases the risk of secondary immune reactions to wear debris (i.e., aseptic osteolysis) post TJA, leading to revision surgery. Furthermore, to what degree do environmental and genetic factors, such as sex and age, affect innate and adaptive immune reactivity to wear debris?

Previous data has shown that innate immune activation of the NLRP3 inflammasome is the trigger that can lead to excessive metal-activated lymphocytes [24]. Specifically, metal implant debris induced inflammasome activation in young wild-type female mice promoted IL-17 producing CD4+ T-cells responses in metal-DTH. It remains unknown how or if this acquired adaptive immune reactivity translates into increased susceptibility of implant failure due to aseptic osteolysis, i.e., bone loss. Further, it remains unknown if sex and/or age influence immune reactivity to implant metals. We hypothesized that both young- and aged-female mice will exhibit higher severity of metal-DTH responses, that consequently translates into increased osteolysis to cobalt-alloy (CoCrMo) particulate debris. To test this, we induced a condition of metal-DTH in both young and aged male and female C57BL/6 mice over the course of 14 days. After the establishment of metal-DTH responses, CoCrMo-implant particles were administrated onto their calvaria for a total of 7 days of particle exposure. At the end of particle challenge the mouse calvaria were retrieved to measure both osteolysis and DTH responses via lymphocyte transformation testing (LTT) and cytokine production. The results of this investigation partially supported our hypothesis. Our investigation showed that in general, younger females are the most susceptible to excessive immune reactivity to implant debris. These results are concerning given the decreasing age of TJA recipients and the overall increasing number of TJAs performed each year [1].

## Materials and methods

### Animal research

Young and aged male and female C57BL/6 (BL/6) and Caspase-1-/- (C57BL/6 background) mice were obtained from The Jackson Laboratory (Bar Harbor, Maine). Two sets of independent experiments were performed with n = 4–5 mice per group age 12–16 weeks or age 18–24 months. Mice were provided a standard laboratory diet and water and maintained under pathogen-free conditions under a 12-hour light/dark cycle. All experiments were carried out under the guidelines of the Institutional Animal Care and Use committee at Rush University Medical Center and approved by the committee on the Ethics of Animal Experiments of Rush University Medical Center (IACUC) and all experiments in our study were conducted adhering to the institution’s guidelines for animal husbandry, and followed the guidelines and basic principals in the Public Health Service Policy on Humane Care and Use of Laboratory Animals, and the Guide for the Care and Use of Laboratory Animals, United States Institute of Laboratory Animal Resources, National Research Council. All efforts were made to minimize suffering; all manipulations were performed under isoflurane anesthesia and mice were sacrificed by cervical dislocation after being anesthetized.

### Media and reagents

Isolated murine lymphocytic cells were cultured with sterile RPMI 1640 supplemented with L-Glutamine, Penicillin, Streptomycin, 25mM Hepes (Lonza, Walkersville, MD) containing 10% heat inactivated fetal bovine serum (FBS; ATCC, Manassas, VA).

Cobalt (II) chloride (CoCl_2_; Co), Ni (II) chloride (NiCl_2_; Ni) phorbol 12-myristate 13-acetate (PMA), ionomycin, phytohemagglutinin (PHA), (Sigma Aldrich, St. Louis, MO) and Complete Freund’s adjuvant (CFA) (InvivoGen, San Diego, CA) were used as challenge agents. NiCl_2_ and CoCl_2_ were reconstituted in sterile water, sterile filtered and freshly prepared for each in vitro and in vivo experiment.

Commercially available Cobalt-alloy particles were purchased from BioEngineering Solutions Inc. (Chicago, IL). These particles were produced from a commercially available Cobalt alloy total hip arthroplasty head component (ZimaloyTM), using a proprietary cryomilling technique. The particle size of Cobalt-alloy (CoCrMo-alloy: 60% Cobalt (Co), 28% Chromium (Cr), <6% Molydbenum (Mo), <1% Ni (Ni), ASTM F75) was characterized by using Scanning Electron Microscopy (SEM) and low angle laser light scattering (LALLS). The median diameter of Cobalt-alloy was 0.36μm ECD using SEM and 0.46μm ECD using LALLS, range 0.15–46 μm ECD, Median aspect ratio = 1.45 and granular in shape (BioEngineering Solutions Inc., Oak Park, IL). This size of particulate debris has been shown to be clinically relevant and able to induce inflammatory responses in immune cells [21, 57, 58]. Particle were cleaned, sterilized, and tested for endotoxin levels before use in experiments (<0.01 eU, Kinetic QCL, BioEngineering Solutions Inc, Oak Park, IL) [59]. Particles were re-suspended in sterile PBS-1x at a concentration of 2 mg/100 μl.

### In vivo induction of Delayed-Type Hypersensitivity (DTH) to orthopedic implant metal(s)

**Fig 1:**
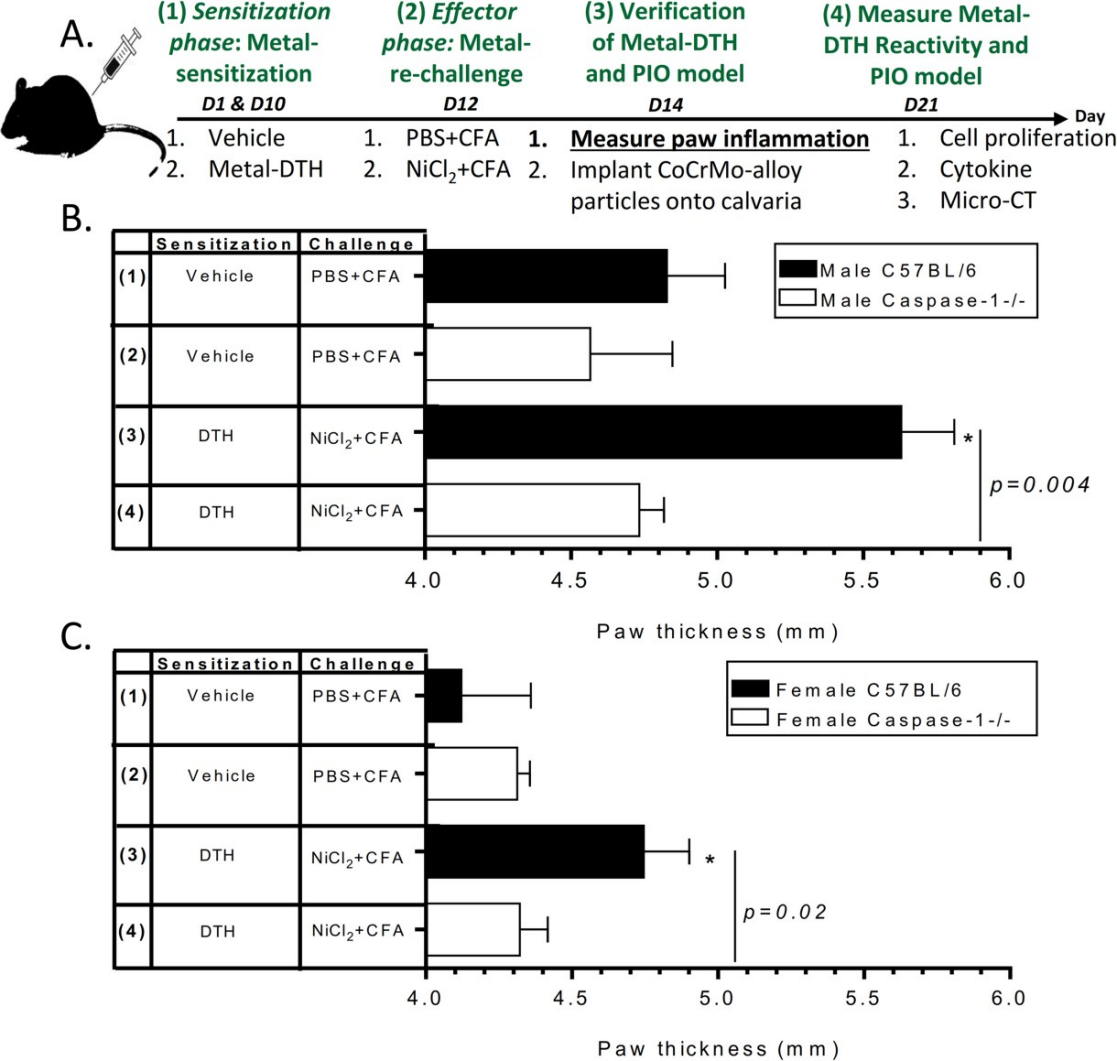
Inflammasome deficient mice are resistant to developing DTH responses to implant metals as determined by paw inflammation to metal challenge. (A) Schematic model for generating metal-DTH to implant metal(s) and particle-induced osteolysis in vivo over 21 days. Metal-DTH responses were determined by measuring paw thickness/inflammation 48 h post challenge on day 14 in both (B) male and (C) female C57BL/6 and Caspase-1-/- mice aged 12–16 weeks that were either non-sensitized (vehicle group) or metal-sensitized (DTH group). Data represent two independent experiments with n = 4–5 mice/group in each experiment. Paw inflammation is represented as the mean ± SEM. Statistical significance was determined by unpaired Mann-Whitney test and asterisk (*) denote significant differences at p < 0.05 compared to respective vehicle group.

To establish a pre-existing condition of Type IV delayed-type hypersensitivity (DTH) to orthopedic implant metal(s) (metal-DTH), we sensitized mice following our previous published protocol (**Fig 1A**) over a 14-day period [24]. Briefly, DTH responses are induced by two phases: 1) sensitization (day 1 and 10) and 2) effector phase (day 12 and 14). To establish sensitization to implant metal(s) (metal-DTH), mice were immunized via intraperitoneal (i.p.) route with either an emulsion cocktail of sterile PBS-1x and adjuvant CFA (vehicle group) at equivolume or with soluble 10mM stock concentration (previously shown to be the most effective concentration for sensitization [60, 61]) of freshly sterile prepared NiCl_2_ and CoCl_2_ and CFA (metal-DTH group) in 125 μl on day 1 and day 10. Mice were boosted/re-exposed to implant metal(s) at day 12 to elicit a DTH reaction in the effector phase. On day 12, mice were boosted via subcutaneous (s.c.) route on top of their right paw with an emulsion cocktail of either sterile PBS-1x (vehicle group) or NiCl_2_ (metal-DTH group) with equivolume of CFA in 50 μl. Induction of local metal-DTH was monitored over 48 hours by measuring right paw inflammation/swelling using a digital caliper in a blinded manner. Each group consisted of n = 4–5 mice and in vivo experiments were performed at two independent times.

### Murine calvaria model of particle-induced osteolysis

On day 14, post induction of a condition of metal-DTH, all mice groups (vehicle and metal-DTH) received CoCrMo-alloy particles onto their calvaria to determine if a pre-existing condition of metal-DTH exacerbates local tissue inflammatory responses (i.e., particle-induced osteolysis (PIO)) upon exposure to implant metal debris. Mice received 2 mg of endotoxin-free CoCrMo-alloy onto the calvaria. Particles were placed on the midline of the calvaria, approximately 1 mm anterior to the line connecting the ears.

### Micro-computed tomography analysis

Osteolysis was determined at 7 days post‐op (receipt) of CoCrMo-alloy particle challenge. To measure the degree of particle-induced osteolysis (PIO), mouse calvaria were harvested, cleaned, and fixed in paraformaldehyde prior to micro-computed tomography (microCT) analysis as previously described (**Fig 1A**), [25, 59, 62]. Briefly, isolated calvaria were gently cleaned of any metallic particles that could act to interfere with the microCT and scanned axially at 55kVp, intensity 145μA, 300-ms integration time with 30-μm isotropic voxels (Scano μCT 40, Wayne, PA, USA). To determine the region of interest (ROI), a three-dimensional rendering of the whole sample was performed using the manufacturer´s software (Amira 5.2 software: TGS, Mercury Computer Systems, Inc., San Diego, CA). Regions of Interest Scans were conducted at a resolution of 9 microns and all portions of the calvaria were included to insure capture of any off-center areas of osteolysis. The top view images of each calvaria sample were analyzed using ImageJ image analysis software to determine % areal osteolysis within a circular control area 1cm in diameter located medially over the central-medial suture lines of the calvaria.

### Lymphocyte Transformation Test (LTT) and cytokine production

To determine the severity of systemically induced metal-DTH reactivity, we performed a lymphocyte transformation test (LTT) as previously described [24]. Briefly, spleens were obtained aseptically at the time of sacrifice on day 21 from vehicle and metal-DTH mice and transferred into a sterile culture dish with 5 mL of PBS with 1mM EDTA. A sterile glass plunger was used to mince/homogenize the spleen to release the splenocytes. Subsequently, to prepare a single-cell suspension splenocytes were collected and passed through a 70 μm strainer on a sterile 50 mL conical tube. Strainer was washed a total of two times with 3 mL of medium. The 50 mL tube containing the cell suspension was centrifuged at 300 x g for total of 10 minutes. To remove red blood cells, cell pellet was processed with Mouse Erythrocyte Lysing Kit (R&D systems; catalog #WL20000) according to manufacturer’s instructions. Cells were washed with wash buffer from the lysing kit and spun down. Splenocytes were re-suspended in media and cell viability was determined using standard trypan blue exclusion test. Isolated splenocytes (composed of lymphocytes, macrophages, and dendritic cells) (5.0X10^5^/well) were cultured with increasing concentrations of soluble nickel (NiCl_2_; Ni) and cobalt (CoCl_2_; Co) at 0.001mM and 0.01mM concentrations for 4 days. Lymphocyte cell proliferation was measured by the incorporation of ^3^H-thymidine for the final 24 hours as described in previously [24]. Supernatants were harvested for cytokine detection by enzyme-linked immunosorbent assay (sandwich ELISAs for mouse IL-17A/F and IFN-gamma; R&D Systems. Inc., Minneapolis, MN).

### Statistical analysis

D’Agostino and Pearson omnibus normality test was performed to determine normality for each of the data sets. If the data set passed the normality test (alpha = 0.05), then data was subsequently analyzed using Fisher’s LSD for comparison of groups post-hoc after significance was determined with ANOVA and is represented as the mean (+SEM). To determine statistically significant differences among intragroup comparisons, two-tailed paired Student’s t-test was used and for intergroup comparisons, unpaired Student’s t-test was used as appropriate. Mann-Whitney was used for the comparison of two groups and Kruskal-Wallis for the comparison of more than two data sets for non-normal distributed data and/or n<15 data points [63]. All statistical analysis was performed using Prism 6.0 program (GraphPad, San Diego, CA). Statistical difference was considered significant at p ≤ 0.05.

## Results

### Male and female BL/6 mice induced DTH reactions to implant metal(s) under controlled environmental conditions

Recent evidence has demonstrated that the incidence and severity of metal sensitization in TJA patients is increased compared to the general population, indicating an association with immune reactions to increased exposure to metals [64]. To determine if NLRP3 inflammasome activation and/or biological sex influence immune metal-DTH responses, we metal-sensitized male and female C57BL/6 (BL/6) vs. Caspase-1-/- mice aged 12–16 weeks under controlled environmental conditions (**Fig 1A**) [24]. Mice were re-exposed to metal allergen on their hind paw on day 12 and on day 14, and localized paw thickness/inflammation was measured in a blindly fashion to assess the severity of metal-DTH response (**Fig 1B and 1C**). Male and female BL/6 metal-DTH mice exhibited significantly increased paw inflammation vs respective controls (**Fig 1B and 1C**), i.e., Male BL/6 metal-DTH p = 0.01; female metal-DTH BL/6 mice p = 0.04). Both male and female Caspase-1 -/- metal-DTH mice however, had significantly less metal-DTH paw inflammation responses when compared to metal-DTH BL/6 mice (respectively p = 0.004 and p = 0.02; **Fig 1B and 1C**). Both male and female wild-type mice were equally susceptible to developing paw inflammation DTH responses to implant metal degradation products, which was dependent on having functional NLRP3 inflammasome/Caspase-1 activity.

### Sex associated inflammasome activation affects lymphocyte reactivity measures of metal-DTH

**Fig 2:**
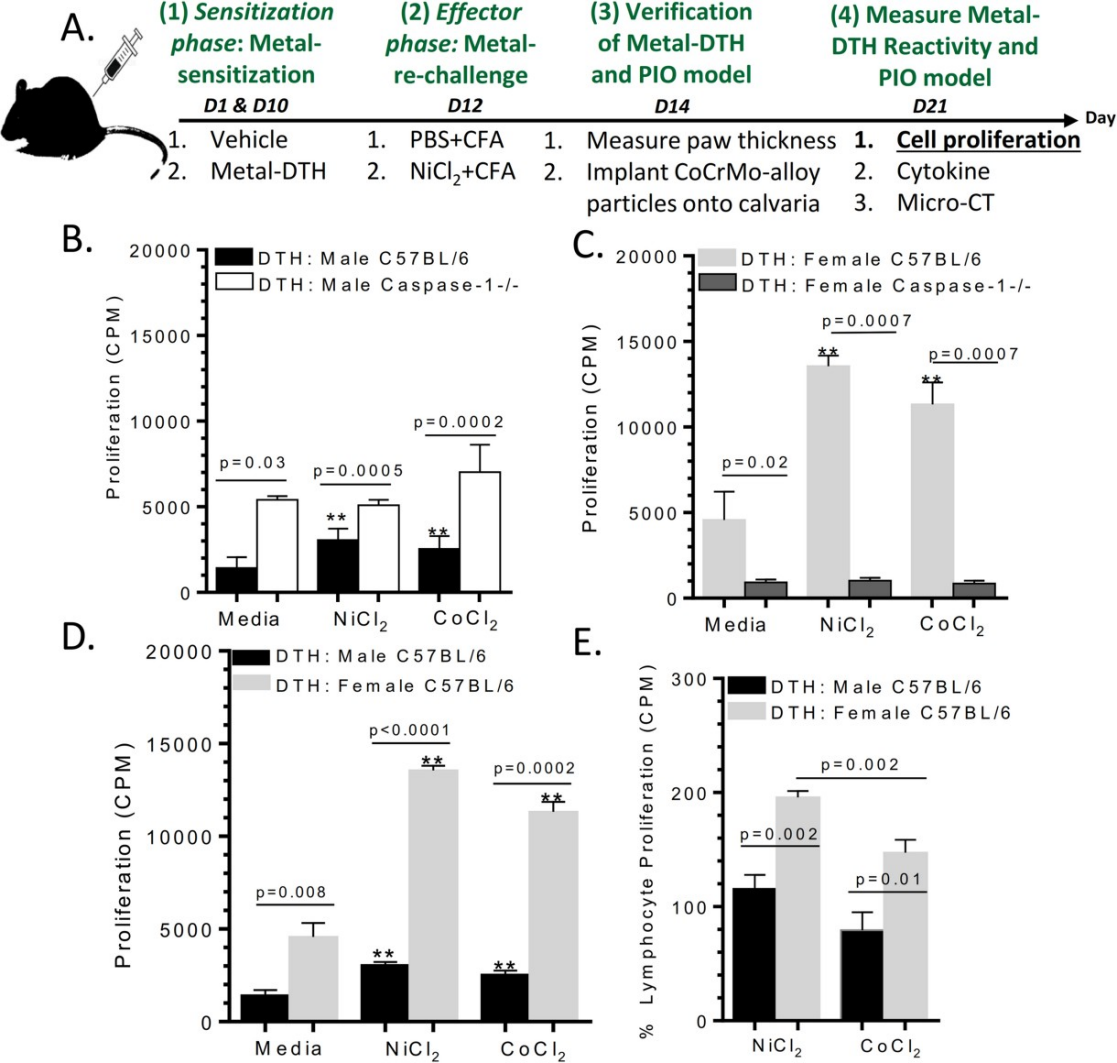
Female metal-sensitized (metal-DTH) BL/6 mice exhibit higher levels of lymphocyte proliferation reactivity. (A) Schematic model demonstrating experimental design. Lymphocytes were purified from mouse spleens at Day 21 and co-cultured with or without Ni or Co challenge for four days to measure amount of lymphocyte proliferation responses via LTT from either (B) male or (C) female metal-DTH BL/6 and Caspase-1 -/- mice, and cell proliferation was measured by ^3^H-thymidine incorporation. (D) Comparison of lymphocyte metal sensitivity responses in male vs. female BL/6 mice, and (E) percentage increase of lymphocyte proliferation to metal challenge; calculated using cell proliferation to metal(s) vs. respective cell proliferation to media. Data represents two independent experiments with n = 4–5 mice/group in each experiment. Data are shown as the mean ± SEM. Statistical significance was determined by unpaired Mann-Whitney test and asterisk (**) denote significant differences at p < 0.005 compared to respective in vitro media challenged cell group.

Metal reactivity to implant metals was further assessed by an in vitro lymphocytic transformation test (LTT) (**Fig 2A**) [24]. Male metal-DTH BL/6 mice demonstrated lymphocyte reactivity to both Ni (p = 0.0001) and Co (p = 0.0005; **Fig 2B**). Male metal-DTH Caspase-1 -/- mice did not exhibit lymphocyte metal reactivity to either Ni or Co in vitro re-stimulation (**Fig 2B**). Female metal-DTH Caspase-1 -/- mice as well did not exhibit increased in vitro lymphocyte metal reactivity (**Fig 2C**). Significant increases in lymphocyte metal responses to both Ni and Co (p = 0.0004) were observed in the female metal-DTH BL/6 group (**Fig 2C**). Additionally, increases in lymphocyte metal reactivity was significantly greater in female metal-DTH BL/6 mice compared with male BL/6 to both Ni (p = <0.0001) and Co (p = 0.0002; **Fig 2D**). These marked differences between male and female mice were further evident when compared on a normalized basis to their respective controls, where the percentage increase of lymphocyte proliferation to metal-challenge was higher in female mice (i.e., Ni (p = 0.002) and Co (p = 0.01) vs. males, **Fig 2E)**. These data further support that metal DTH is centrally dependent on inflammasome/caspase-1 biological activity as a gatekeeper. These results indicate that female BL/6 mice exhibit more severe immune reactions i.e., metal hypersensitivity to implant metals Ni and Co compared with males when both have been exposed to identical environmental conditions.

### Metal-sensitized female BL/6 mice preferentially exhibit IL-17A/F production

**Fig 3:**
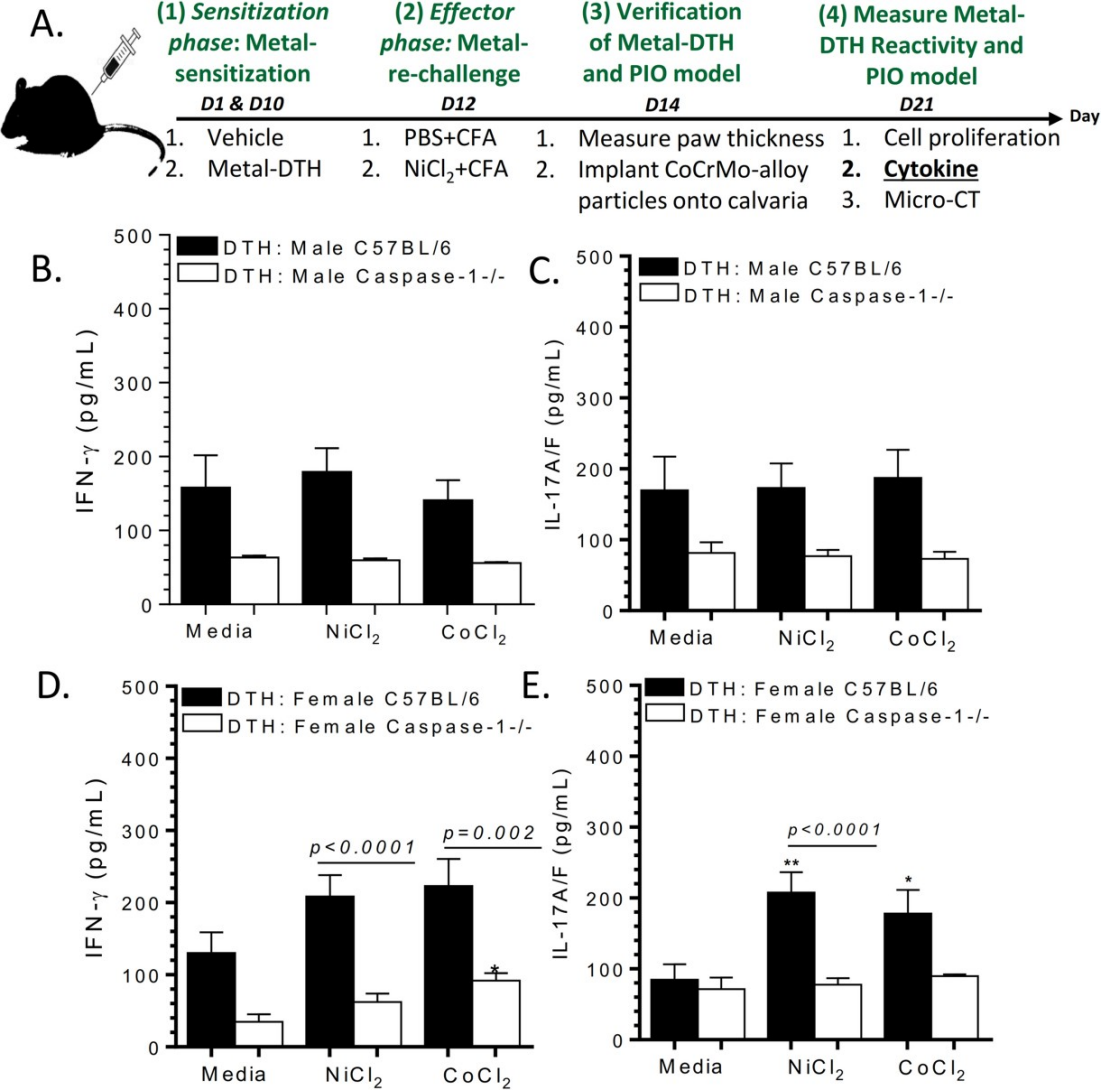
Female vs male BL/6 metal-DTH mice exhibit preferential IL-17A/F producing T-cells to implant metal-challenge. (A) Schematic model demonstrating experimental design. At day 21, spleens were harvested. Lymphocytes were purified from mouse spleens and co-cultured with or without Ni or Co challenge for four days and supernatants were collected and analyzed for cytokine production. Lymphocytic (B) IFN-gamma and (C) IL-17A/F production was measured from metal-DTH treated male BL/6 and Caspase-1 -/-. Lymphocytic (D) IFN-gamma and (E) IL-17A/F production was also measured from metal-DTH treated female BL/6 and Caspase-1 -/- mice. Data represents two independent experiments with n = 4–5 mice/group in each experiment. Data are shown as the mean ± SEM. Statistical significance was determined by unpaired Mann-Whitney test and asterisk (**) denote significant differences at p < 0.005 and (*) p < 0.05 compared to respective in vitro media challenged cell group.

To further assess metal-activated lymphocyte responses, we analyzed production of pro-inflammatory T-cell cytokines such as IL-17A/F and IFN-gamma upon re-stimulation with implant metal(s) **(Fig 3A)**. The modest increase in lymphocyte proliferative responses from metal-DTH male BL/6 mice did not correspond with any significant cytokine production of either IFN-gamma or IL-17A/F (**Fig 3B and 3C**). Metal-sensitized Caspase-1 knockout (metal-DTH Caspase-1-/-) male mice also did not exhibit any increases in lymphocyte cytokine production to metal challenge (**Fig 3B and 3C**). Similarly, metal-DTH female BL/6 mice did not exhibit any significant differences in amount of lymphocytic IFN-gamma production to metal-challenge (**Fig 3D**). However, female Caspsase-1 -/- metal-DTH treated mice demonstrated an increase in IFN-gamma production in response to Co challenge (p = 0.04; **Fig 3D**). And more importantly, female BL/6 mice exhibited significant lymphocytic production of IL-17A/F in response to both Ni (p = 0.002) and Co (p = 0.03; **Fig 3E**).

**Fig 4:**
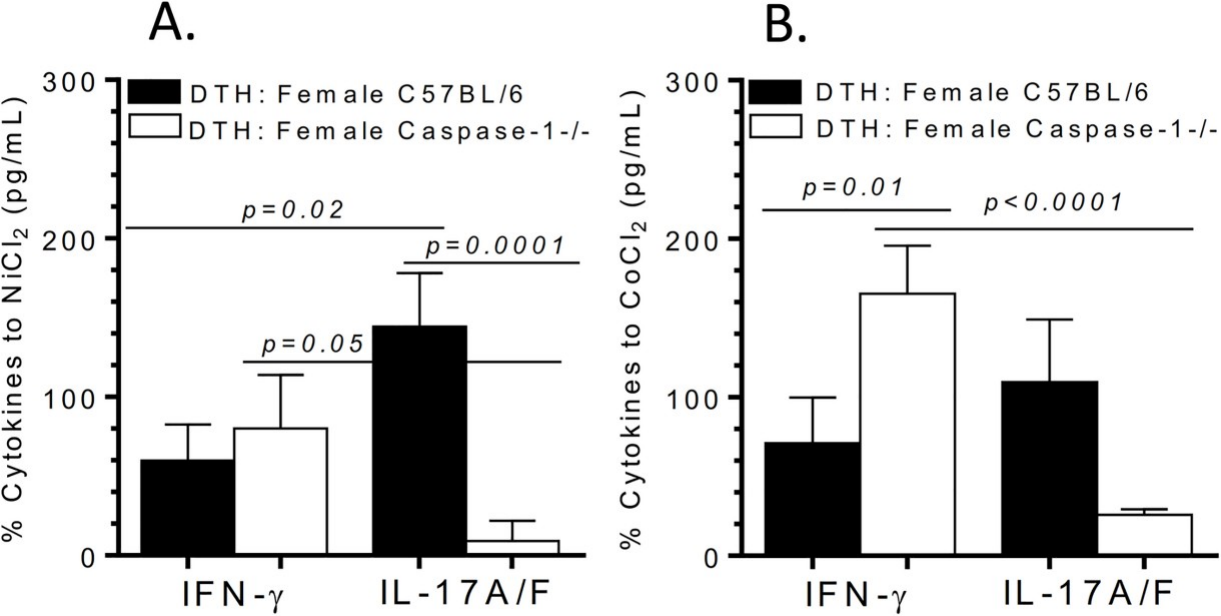
Metal-sensitized caspase-1 knockout female (DTH: Female caspase-1 -/-) mice produce IFN-gamma dominated T-cell responses to implant metal-challenge. Percentage increase of lymphocyte cytokine production of IFN-gamma or IL-17A/F in response to (A) Ni challenge and (B) Co challenge; calculated using cytokine production to metal(s) vs. respective negative control media cell cytokine production from female metal-DTH BL/6 and Caspase-1 -/- mice. Data represents two independent experiments with n = 4–5 mice/group in each experiment. Data are shown as the mean ± SEM. Statistical significance was determined by unpaired Mann-Whitney test.

Additionally, metal-sensitized females (metal-DTH BL/6) not only exhibited greater in vitro metal proliferative reactivity compared with males (metal-DTH BL/6), but also demonstrated more IL-17A/F production to Ni (144.37 pg/mL) than IFN-gamma (59.95 pg/mL; p = 0.02; **Fig 4A**). IL-17A/F production to Ni by female BL/6 mice was significantly greater than IL-17A/F observed in Caspase-1-/- female mice (p = 0.0001). While female Caspase-1 -/- isolated lymphocytes produced increased IFN-gamma to Ni and Co challenge compared to respective IL-17A/F production (**Fig 4A and 4B**), it did not correspondingly result in increased lymphocyte proliferation responses as reported above (**Fig 2**).

**Fig 5:**
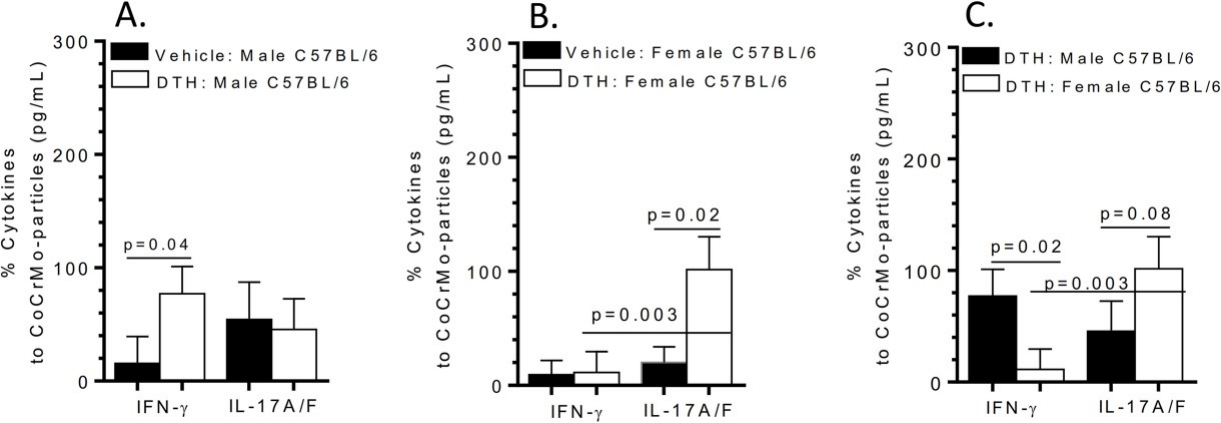
Female vs male BL/6 lymphocyte cytokine responses to CoCrMo-alloy particle challenge. Percentage increase of lymphocyte cytokine production of IFN-gamma or IL-17A/F in response to CoCrMo-alloy particle challenge from either vehicle or metal-DTH (A) male BL/6 or (B) female BL/6 mice; calculated using cytokine production to CoCrMo-alloy particle vs. respective negative control media cell cytokine production. (C) Comparison of male vs. female metal-DTH lymphocytic cytokine responses to CoCrMo-alloy particle challenge. Data represents two independent experiments with n = 4–5 mice/group in each experiment. Data are shown as the mean ± SEM. Statistical significance was determined by unpaired Mann-Whitney test.

Metal-DTH male BL/6 mice produced IFN-gamma dominated lymphocytic responses to CoCrMo-alloy particles challenge (**Fig 5A**), while female metal-DTH BL/6 mice skewed towards IL-17 production when challenged with CoCrMo-alloy particles (**Fig 5B**). These results continue to support sex-differences in lymphocyte metal sensitivity responses (**Fig 5C**).

### Metal-DTH augments particle-induced osteolysis

**Fig 6:**
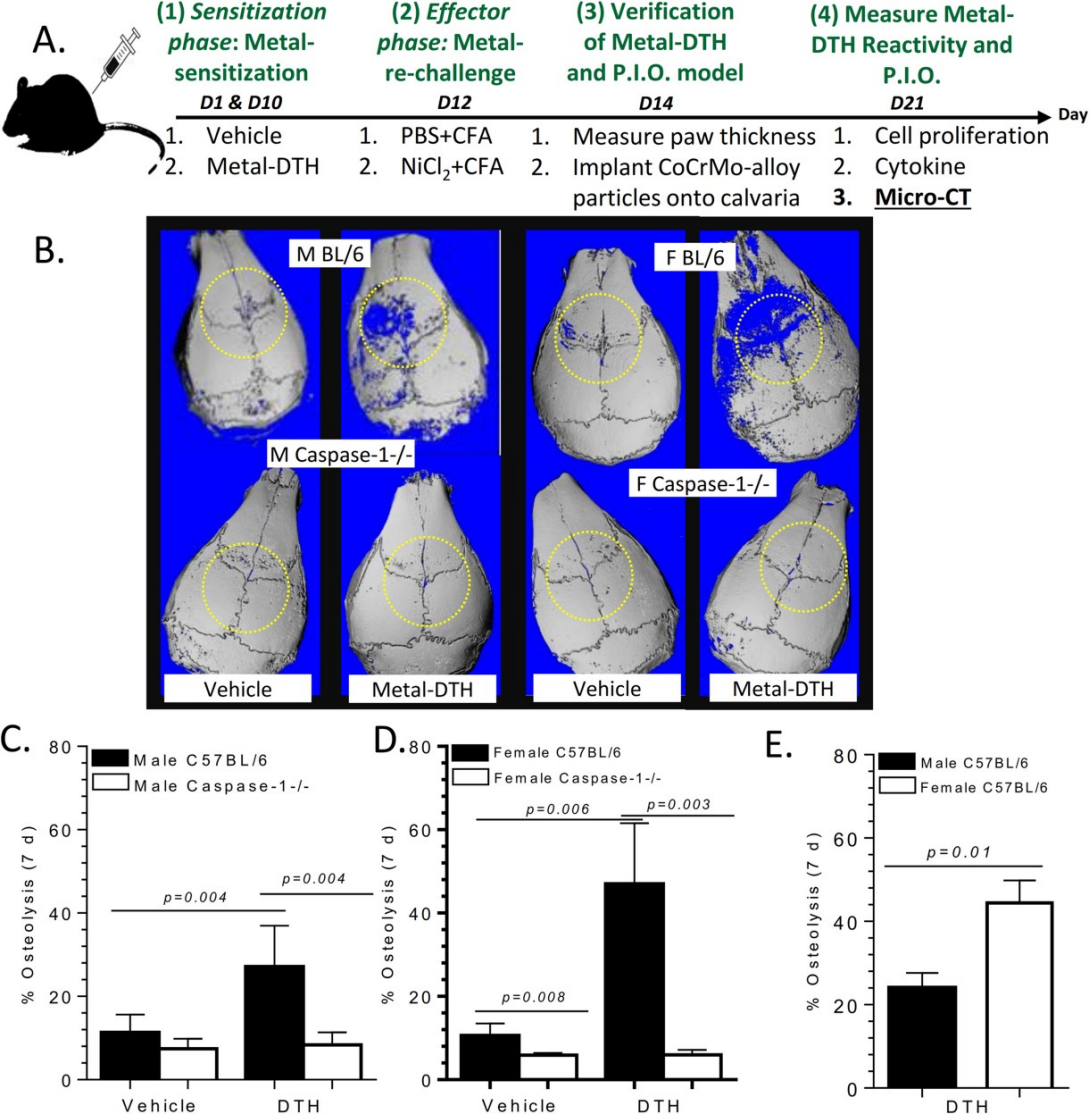
A pre-existing condition of metal-DTH augments metal particle-induced osteolysis. (A) Schematic model demonstrating experimental design where both vehicle and metal-DTH treated male and female C57BL/6 and Caspase-1 -/- mice received 2 mg/mouse calvaria of endotoxin-free CoCrMo-alloy particles. 7 days post-particle implantation (on day 21), calvaria were retrieved and analyzed by microCT. (B) Representative images and graphical representation of the percentage of particle-induced osteolysis from vehicle and metal-DTH treated (C) male and (D) female BL/6 and Caspase-1 -/- mice. (E) Comparison of percentage of bone loss from metal-DTH treated male vs. female BL/6 mice. Data represents two independent experiments with n = 4–5 mice/group in each experiment. Data are shown as the mean ± SEM. Statistical significance was determined by unpaired Mann-Whitney test.

Does a condition of metal-induced DTH result in increased particle-induced osteolysis (PIO) (**Fig 6A**)? We determined if a pre-existing condition of adaptive immunity to specific metal exposure is a factor that may influence sex-based differences in particle induced osteolysis outcome. DTH mice groups on day 14, post vehicle or metal-sensitization treatment, received subcutaneous placement of 2mg of endotoxin-free CoCrMo-alloy particles onto their calvaria (**Fig 6A**). The amount of calvaria osteolysis was measured 7 days post-particle implantation (on day 21). Both male and female metal-DTH BL/6 mice exhibited significant bone loss (**Fig 6B–6D**). In contrast, both male and female Caspase-1-/- mice were protected from developing PIO. Additionally, female metal-DTH BL/6 mice exhibited significantly more PIO than male metal-DTH BL/6 mice (p = 0.01; **Fig 6E**). These results demonstrate that: 1) a pre-existing condition of DTH to implant metal(s) can augment PIO, 2) female wild-type mice are more susceptible than male wild-type mice to metal-DTH augmented PIO, and 3) active inflammasome/caspase-1 pathway is a required immune response to implant metals that allow metal-DTH augmented PIO.

### Comparison of metal-DTH lymphocyte responses between young and aged male and female BL/6 mice

**Fig 7:**
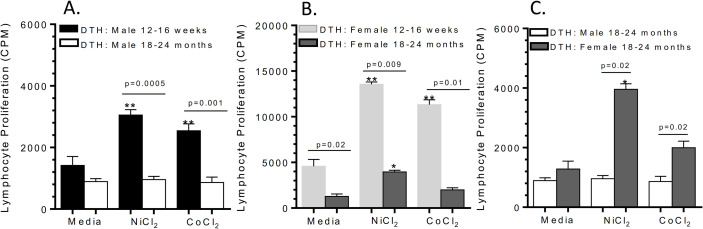
Age and sex effects lymphocyte metal-DTH responses. Post metal-DTH induction, lymphocytes were purified from mouse spleens and co-cultured with or without Ni or Co challenge. Comparison of lymphocyte proliferation to Ni and Co from metal-DTH younger and aged: (A) males, (B) females. And comparison of LTT responses among (C) aged male vs. female metal-DTH treated BL/6 mice. Cell proliferation was measured by ^3^H-thymidine incorporation. Data represents two independent experiments with n = 4–5 mice/group in each experiment. Data are shown as the mean ± SEM. Statistical significance was determined by unpaired Mann-Whitney test and asterisk (**) denote significant differences at p < 0.005 and (*) p < 0.05 compared to respective in vitro media challenged cell group.

To determine if age is a significant factor in metal-DTH augmented PIO, male and female BL/6 mice aged 18–24 months were metal-sensitized following the same procedure as previously outlined. On day 21, we measured lymphocyte proliferative responses to metal challenge as measured by quantitative LTT. Aged metal-DTH male mice did not exhibit increased lymphocyte metal-reactivity (**Fig 7A**). In contrast, aged metal-DTH female mice demonstrated significant increase lymphocyte proliferation to Ni (p = 0.03) but not to Co challenge (**Fig 7B**). However, younger female mice exhibited significantly higher lymphocyte proliferation responses to both Ni (p = 0.009) and Co (p = 0.01) when compared to aged female mice (**Fig 7B**). Aged female metal-DTH mice demonstrated increased lymphocyte reactivity to Ni (p = 0.02) when compared to respective aged male metal-DTH mice (**Fig 7C**). Taken together, these results demonstrate that metal-DTH responses are not only influenced by sex, but also by age. Additionally, Ni ion challenge provoked a greater immune response than Co among females of any age.

### IL-17 production to implant metals is reduced in aged female (vs young) metal-DTH mice

**Fig 8:**
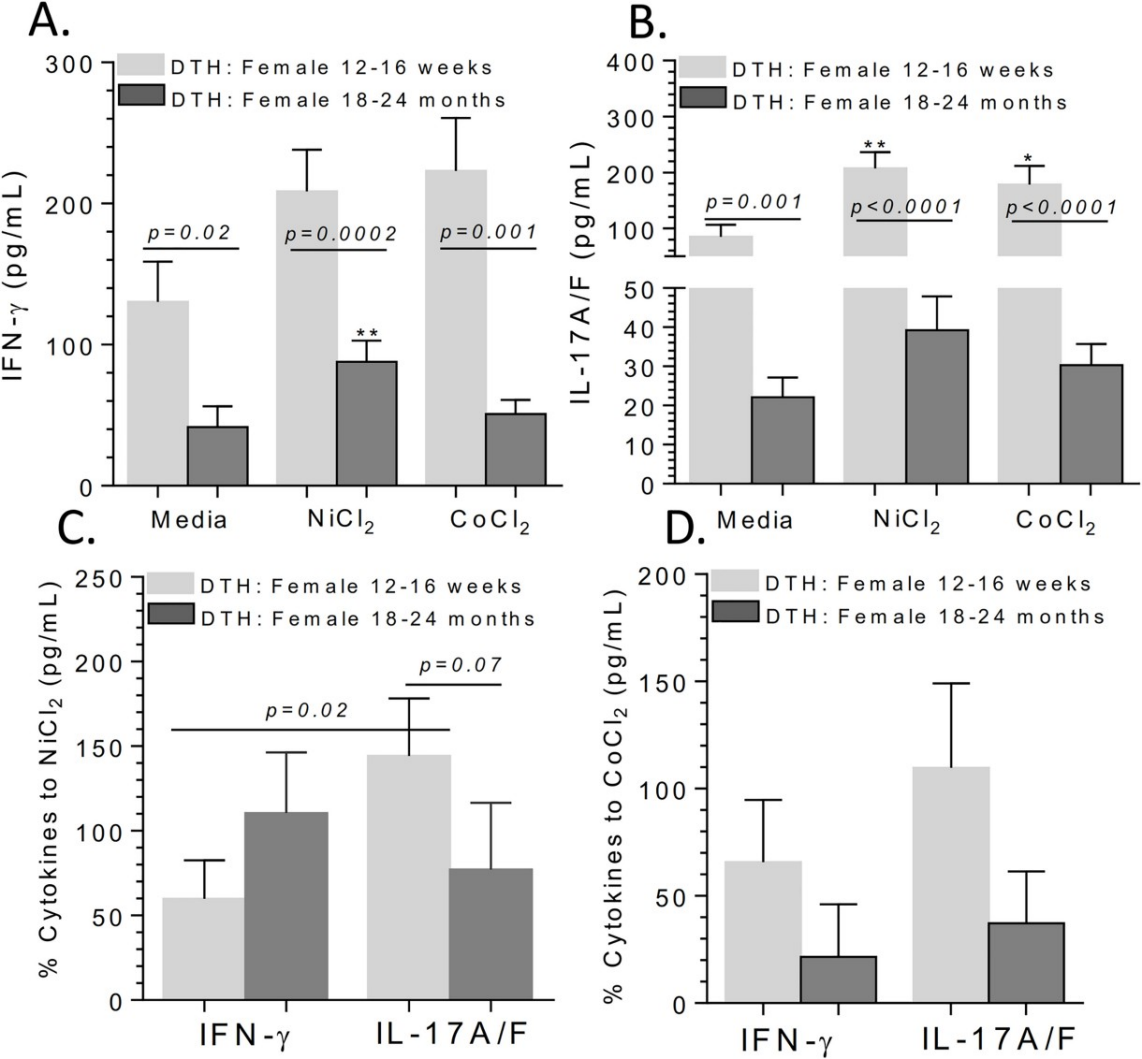
IL-17 production is reduced in aged (vs young) metal-DTH female mice. Lymphocytes were restimulated in vitro with soluble metal ions for 4 days and (A) IFN-gamma and (B) IL-17A/F production were measured from supernatants by ELISA. Percentage increase of lymphocyte cytokine production of IFN-gamma or IL-17A/F in response to (C) Ni or (C) Co metal ion challenge; calculated using cytokine production to metal ions vs. respective negative control media cell cytokine production. Data represents two independent experiments with n = 4–5 mice/group in each experiment. Data are shown as the mean ± SEM. Statistical significance was determined by unpaired Mann-Whitney test.

To determine whether the reduction in metal-specific T-cell proliferation corresponds to reduction in T-cell cytokine secretion, we assessed IFN-gamma and IL-17A/F production from aged mice. The quantity of IFN-gamma and IL-17 production was markedly lower in metal-stimulated lymphocytes from aged male vehicle and metal-DTH treated mice (**S6 and S7 Tables**). We observed notable differences in IFN-gamma production to only Ni- stimulated lymphocytes from aged female metal-DTH mice (p = 0.003; **Fig 8A and 8B**). Younger female metal-DTH mice demonstrated IL-17 mediated immune responses (**Fig 8C and 8D)**, without IFN-gamma production in response to Ni and Co challenge. The amount of IFN-gamma production to Ni was approximately 2-fold higher in aged vs. younger female metal-DTH mice (**Fig 8C**). Conversely, there was an approximately 2-fold increase in the amount of IL-17 production to Ni in younger vs. aged mice, and there was a 2.4-fold increase in the amount of IL-17 compared to IFN-gamma production to Ni in young female mice (p = 0.02; **Fig 8C**). Thus, the reduction in IL-17 production with concomitant relative increase in IFN-gamma T-cell responses in aged female metal-DTH mice correlated with decreased degree of severity of lymphocyte metal reactivity/proliferation.

### Comparison of metal-DTH augmented particle induced osteolysis (PIO) between young and aged male and female mice

**Fig 9:**
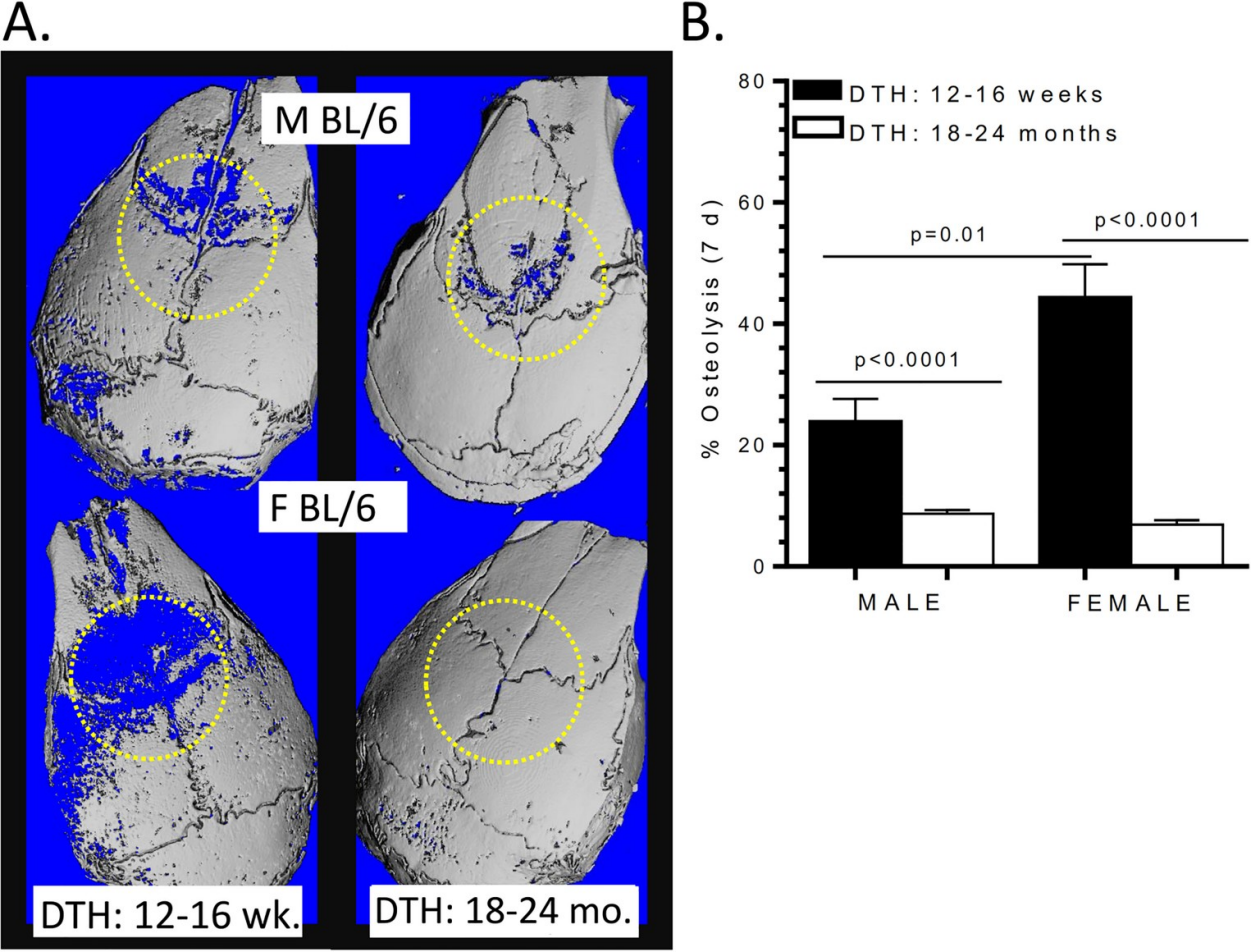
Young female mice are more susceptible to experiencing metal-induced PIO. Metal-DTH treated young and aged male and female BL/6 mice received 2 mg/mouse calvaria of endotoxin-free CoCrMo-alloy particles. 7 days post-particle implantation (on day 21), calvaria were retrieved and analyzed by microCT. (A) Representative images and (B) graphical representation of the percentage of particle-induced osteolysis. Data represents two independent experiments with n = 4–5 mice/group in each experiment. Data are shown as the mean ± SEM. Statistical significance was determined by unpaired Mann-Whitney test.

We observed no significant differences in the amount of metal-DTH induced PIO between aged male and female mice (**Fig 9**). In contrast, CoCrMo-alloy particles induced more PIO in young male mice compared to aged males (p = <0.0001; **Fig 9B**). However, metal-DTH induced PIO caused significantly more bone loss among younger female mice than any other group tested (**Fig 9B**). This data indicates that younger aged female mice are more vulnerable to experiencing metal-DTH responses that leads to exacerbated secondary immune inflammatory responses to wear debris, resulting in increased PIO. Thus, there is a correlation between the level of T-cell proliferation, IL-17 responses, and the degree of bone loss.

## Discussion

It is unclear to what degree a developed adaptive immune response to implant metal(s) contribute to implant debris mediated pain and osteolysis leading to biological implant failure (BIF), i.e., revision surgery. Thus, this study employed the biologic complexity of an in vivo model to determine if a pre-existing condition of metal DTH prior to an individual receiving a primary TJR, increases risk of adverse local tissue inflammatory reactions to metal implant debris (i.e., aseptic osteolysis) post TJR in specific patient populations. In the present study, we provide evidence that supports active inflammasome/caspase-1 dependent IL-1 signaling is required for the development and generation of metal-specific T-cell immune reactions and as a gatekeeper or checkpoint for DTH augmented osteolysis [24]. A deficiency in inflammasome (i.e., Caspase-1 -/- mice) activity suppresses the development and generation of metal-specific T-cell mediated immune responses as assessed by significantly reduced recall lymphocyte activation/proliferation response to implant metal restimulation in vitro (**Figs 1 and 2**). Consistent with our previous investigation [24], we found that metal-restimulated T-cells from metal-sensitized female BL/6 mice produce increased levels of IL-17 and lower levels of IFN-gamma, whereas T-cells from metal-sensitized female mice without well-functioning inflammasome danger signaling (i.e. Caspase-1 -/- mice) produce the converse, i.e. increased levels of IFN-gamma and lower levels of IL-17 (**Figs 3 and 4**) [24]. In contrast to females, male Caspase-1 -/- metal-DTH mice did not exhibit a severe lymphocytic reaction to metal restimulation in vitro. Not surprisingly however, despite an increase in IFN-gamma production to implant metals by female Caspase1 -/- mice, there was no significant increase in the degree of bone loss. Instead, both female and male Caspase-1 -/- mice were effectively protected from developing metal-DTH responses as well as secondary local tissue reactions to metal debris as determined by bone loss. Since it is known that cytokines produced by innate immune cells such as IL-1β regulate the induction and expansion of IL-17 producing CD4+ T cells, it was expected that a deficiency in inflammasome/capase-1 would suppresses the differentiation of Th17 cells, i.e., production of IL-17, as was found.

It has been recently reported that female TJA patients with idiopathic joint pain have significantly increased rate and severity of lymphocyte metal sensitivity responses as determined by metal-LTT compared to male TJA patients [47]. Our data supports this correlation as causational when using an in vivo murine model of induced DTH and PIO, where both young male and female BL/6 mice with an induced/established condition of metal sensitivity, were found more susceptible to inflammatory tissue reactions to metal wear particles, which also translated into significantly increased amounts of aseptic osteolysis. However, male mice exhibited a lower level of metal-specific T-cell recall proliferation (**Fig 2**) and bone loss (**Fig 6**) when compared to age-matched female mice. Metal-DTH female mice (vs males) exhibited an approximately 2-fold increase of lymphocyte reactivity to Ni and Co ion challenge (**Fig 2E**), which directly translated into similar approximately 2-fold increase in the amount of particle induced osteolysis. T-cells from male mice produced very little IL-17 and increased IFN-gamma in response to only CoCrMo-alloy particle challenge. Conversely, female mice preferentially expressed elevated IL-17 and very little IFN-gamma in response to both metal ion and particle challenge (**Figs 4 and 5**). The importance of this difference has been made clear by several autoimmune disease models such as collagen-induced arthritis (CIA) and experimental autoimmune encephalomyelitis (EAE), where ablation of IFN-gamma resulted in exacerbated disease due to increase in production of mature IL-1β and IL-17, as well as enhanced T cell proliferative responses [65]. This has also been shown in a subset of patients where a significant correlation between increased IL-17 and decreased IFN-gamma levels correlated with progression to arthritis and more severe aggressive form of disease [66]. As a result, several IL-17A inhibitors are clinically available and show favorable responses in a subset of arthritis patients, but not all [67].

This study indicates that sex-specific expression of adaptive immune cytokines from metal-stimulated lymphocytes are associated with metal-DTH and local immune and tissue responses to metal particles. Specifically, our current data indicates that young female mice are more vulnerable than males or older female mice, to experiencing a higher intensity of metal-activated lymphocytes that release osteolysis promoting cytokines that exacerbated bone loss to implant debris; and this process is intrinsically linked to innate immune inflammasome induction of adaptive immune system IL-17 expression. The results of this murine study cannot be directly extrapolated to humans. However, if our results are true for larger mammals and humans then younger female orthopedic patients may have a higher risk of DTH type elevated reactivity than men. The driving mechanism behind this may be due to less dominant IFN-gamma and thus more dominant IL-17 lymphocyte responses to wear debris. In the absence of IFN-gamma expressing T-cells, IL-17 expressing T-cells are increased and can drive immune responses to wear debris. Our in vivo murine findings are also consistent with a greater prevalence of autoimmune diseases and arthritis in women than in men [68]. Further study is required to determine if these in vivo murine results are applicable/translate in the clinical setting.

However, this increased immune and bone loss reactivity to metal implant debris in female mice appeared to disappear with age. We found a significant reduction in metal-specific T-cell recall responses in aged metal-sensitized male and female BL/6 mice, which translated into suppressed secondary immuno-inflammatory responses to wear debris, resulting in minimal bone loss. Aged female mice exhibited substantial but not complete reduction in lymphocyte proliferation responses to metal restimulation in vitro (**Fig 7**). Additionally, aged female BL/6 mice exhibited significantly increased production of IFN-gamma and decreased IL-17 when compared to young female mice (**Fig 8**). This age-dependent increase in IFN-gamma production to Ni was like the increase observed in female Caspase-1 -/-. Thus, age related changes in innate immune danger sensing may translate into a less aggressive or altered adaptive immune profiles with a shift from IL-17 to elevated IFN-gamma centric reactivity. Current efforts are underway to further examine this using particle induced osteolysis in aged inflammasome deficient male and female metal-DTH mice.

In general, studies in aged mice (> 20 months) and in humans (> 65 years) have demonstrated that innate immune activation can lead to dysregulated inflammation [69]. The term “inflamm-aging” has been used to describe this phenomenon of age-associated impairment in immune responses due to chronic low-grade inflammation [69]; and in part due to the loss of antigen-specific T-cells (memory and naïve) with age [70]. In contrast, long-lived individuals, specifically centenarians, exhibit relatively more robust anti-inflammatory responses which subdue chronic subclinical inflammation, and is termed “anti-inflammaging” [71]. Thus, inflamm-aging is regarded as a delicate and intricate balance between pro- and anti- innate and adaptive inflammatory responses. Recently, it has been demonstrated that titanium-particle in vitro challenged macrophages from young 2-month-old male C57BL/6 mice expressed increased mRNA expression of cytokines IL-1β, IL-6 and TNF-α compared to aged macrophages from 18-month-old male C57BL/6 mice [72]. While in vitro metal-stimulated macrophages from aged mice produced increased amounts of IL-1 receptor antagonist (IL-1Ra), an anti-inflammatory cytokine that is central to suppressing IL-1β pro-inflammatory activity [72]. It has also been reported aged healthy individuals (> 65 years) compared to young individuals (< 40 years), exhibited a decreased frequency of not only IL-17 producing CD4+ T cells but also a decrease ratio of IL-17 to IFN-gamma producing T cells (i.e. decreased ratio of Th17 to Th1 cells) in peripheral blood [73]. Thus, there appears to be an altered ratio of Th17/Th1 cells associated with aging. However, it is presently unclear, why aged mice in our study are protected from augmented metal-DTH bone loss to metal implant debris. Several possibilities include 1) chronic low-grade inflammation has restricted innate inflammasome immune responsiveness and/or 2) impaired adaptive immune responses, or if 3) aged mice exhibit heightened anti-inflammatory cytokines (i.e., IL-1Ra, IL-10 etc.). Further studies will be needed to address the roles of each of these possibilities.

We acknowledge several limitations to our study. First, the murine calvaria model does not perfectly mimic/translate clinically in terms of implant particle load and daily exposure, as well as the periprosthetic joint space. Secondly, while all efforts are made to implant particles in same location on the mouse calvaria, it is not possible to prevent migration of all particles from site of implantation. This varies from calvaria to calvaria to some extent and can cause differences in generalized inflammation and bone responses such as thinning outside the established inoculation site. Additionally, the threshold values for soft and hard tissue on microCT may differ dependent on the type and quality of bone and microCT machine; however, these values were constant for all groups tested and showed differences for established calvaria bone/hard tissue not inclusive of non-mineralized neogenic bone. Despite this however, this animal model has been widely used and is easily reproducible among groups. Another important limitation of this study is that practical limitations restricted analysis to single time point for lymphocyte function and bone loss measurement. While there are minimal studies reporting the effect of age on PIO, these investigations have shown that PIO in younger mice peaked at day 7, while PIO in older mice peaked at later time points such a day 14 and day 21 [74, 75]. These studies differ from the current investigation however, in terms of the type of particle used (i.e., UHMWPE [74] or pure titantium particles [75]), as well as the strain and sex of the mice. Our study is consistent with these previous reports in that the osteolytic response in younger aged mice is rapid and peaks a day 7 post particle exposure onto the calvaria. Further investigation including longer time-points as well as measuring the local rates of bone loss vs. bone formation in young and aged male and female mice are required and critical to further understanding how sex and/or aging of the immune system plays a role in the response of innate and adaptive immune response to implant wear debris.

Within the limits of this study, our results suggest that younger females with a pre-existing condition of metal sensitivity are more susceptible to excessive immune responses to wear debris post-TJR, which may influence aseptic implant failure rates. More specifically, our results suggest that differential expression of IFN-gamma producing Th1 cells versus IL-17 producing Th17 cells may in part be responsible for resultant metal-DTH augmented particle induced bone loss and is consistent with a relatively higher risk of implant failure among younger females vs older females and all males. These findings are clinically important to understand which patient populations are more predisposed or likely to suffer biologic implant failure and how to combat this therapeutically.

## Supporting information

S1 Table Lymphocyte proliferation from young mice. Mean lymphocyte proliferation expression values + SEM as presented in Fig 2.
(PDF)

S2 Table Lymphocyte IFN-gamma production from young mice. Mean IFN-gamma production expression values + SEM as presented in Fig 3.
(PDF)

S3 Table Lymphocyte IL-17A/F production from young mice. Mean IL-17A/F production expression values + SEM as presented in Fig 3.
(PDF)

S4 Table Percentage of particle-induced osteolysis (P.I.O) from young mice. Mean osteolysis percentage expression values + SEM as presented in Fig 6.
(PDF)

S5 Table Lymphocyte proliferation from aged mice. Mean lymphocyte proliferation expression values + SEM as presented in Fig 7.
(PDF)

S6 Table Lymphocyte IFN-gamma production from aged mice. Mean IFN-gamma production expression values + SEM as presented in Fig 8.
(PDF)

S7 Table Lymphocyte IL-17A/F production from aged mice. Mean IL-17A/F production expression values + SEM as presented in Fig 8.
(PDF)

S8 Table Percentage of particle-induced osteolysis (P.I.O) from aged mice. Mean osteolysis percentage expression values + SEM as presented in Fig 9.
(PDF)
