# Supplementary material for: Metal-induced delayed type hypersensitivity responses potentiate particle induced osteolysis in a sex and age dependent manner
Source: PLoS One. 2021 May 18;16(5):e0251885. doi: 10.1371/journal.pone.0251885 (PMC8130946; doi:10.1371/journal.pone.0251885)
Supplement: S1 Table — Mean lymphocyte proliferation expression values + SEM as presented in Fig 2. (PDF) [file pone.0251885.s001.pdf]

S1 Table: Proliferation (CPM)	*Media*		*$NiCl_2$*		*$CoCl_2$*	
**Group (12-16 weeks old):**	**Mean**	**SEM**	**Mean**	**SEM**	**Mean**	**SEM**
**DTH:M BL/6**	1417	288.5	3055	173.6	2542	224.5
**DTH:M Caspase-1-/-**	5406	125.5	5095	157.3	7019	655.6
**DTH:F BL/6**	4582	736.7	13555	252	11332	861.6
**DTH:F Caspase-1-/-**	920.5	84.84	1032	58.62	519.3	59.22
