# Supplementary material for: Metal-induced delayed type hypersensitivity responses potentiate particle induced osteolysis in a sex and age dependent manner
Source: PLoS One. 2021 May 18;16(5):e0251885. doi: 10.1371/journal.pone.0251885 (PMC8130946; doi:10.1371/journal.pone.0251885)
Supplement: S2 Table — Mean IFN-gamma production expression values + SEM as presented in Fig 3. (PDF) [file pone.0251885.s002.pdf]

S2 Table: IFN-gamma (pg /mL)	*Media*		*$NiCl_2$*		*$CoCl_2$*	
**Group (12-16 weeks old):**	**Mean**	**SEM**	**Mean**	**SEM**	**Mean**	**SEM**
**Vehicle:M BL/6**	295.7	47.73	558.5	40.18	302.9	39.31
**Vehicle:M Caspase-1-/-**	58.59	0.7224	66.62	3.252	60.94	0.8891
**Vehicle:F BL/6**	77.21	7.977	74.81	3.893	75.96	9.187
**Vehicle:F Caspase-1-/-**	56.39	1.566	55.74	1.858	54.49	1.561
**DTH:M BL/6**	158	43.66	179.3	32.1	140.9	55.93
**DTH:M Caspase-1-/-**	63.56	2.481	59.92	2.555	27.06	1.364
**DTH:F BL/6**	130.4	28.44	208.6	29.66	223.2	37.33
**DTH:F Caspase-1-/-**	34.61	10.42	62.38	11.68	91.88	10.49
