# Supplementary material for: Metal-induced delayed type hypersensitivity responses potentiate particle induced osteolysis in a sex and age dependent manner
Source: PLoS One. 2021 May 18;16(5):e0251885. doi: 10.1371/journal.pone.0251885 (PMC8130946; doi:10.1371/journal.pone.0251885)
Supplement: S3 Table — Mean IL-17A/F production expression values + SEM as presented in Fig 3. (PDF) [file pone.0251885.s003.pdf]

S3 Table: IL-17 A/F (pg / mL)	*Media*		*$NiCl_2$*		*$CoCl_2$*	
**Group (12-16 weeks old):**	**Mean**	**SEM**	**Mean**	**SEM**	**Mean**	**SEM**
**Vehicle:M BL/6**	334.8	89.04	304.2	48.12	358.7	67.67
**Vehicle:M Caspase-1-/-**	86.72	16.75	95.74	7.185	76.24	9.349
**Vehicle:F BL/6**	304.5	71.06	272.1	38.37	281.4	40.57
**Vehicle:F Caspase-1-/-**	74.44	14.67	70.09	11.34	70.87	11.1
**DTH:M BL/6**	169.6	47.48	173	34.58	187.5	73.22
**DTH:M Caspase-1-/-**	81.46	15.09	76.79	8.998	39.52	9.766
**DTH:F BL/6**	84.98	21.55	207.7	28.73	178.2	33.39
**DTH:F Caspase-1-/-**	71.36	16.28	77.89	9.083	89.89	2.389
