# Supplementary material for: Metal-induced delayed type hypersensitivity responses potentiate particle induced osteolysis in a sex and age dependent manner
Source: PLoS One. 2021 May 18;16(5):e0251885. doi: 10.1371/journal.pone.0251885 (PMC8130946; doi:10.1371/journal.pone.0251885)
Supplement: S4 Table — Mean osteolysis percentage expression values + SEM as presented in Fig 6. (PDF) [file pone.0251885.s004.pdf]

S4 Table: P.I.O.	*% Osteolysis*	
**Group (12-16 weeks old):**	**Mean**	**SEM**
**Vehicle:M BL/6**	11.38	1.733
**Vehicle:M Caspase-1-/-**	7.456	1.052
**Vehicle:F BL/6**	10.6	1.455
**Vehicle:F Caspase-1-/-**	5.889	0.2377
**DTH:M BL/6**	27.17	3.997
**DTH:M Caspase-1-/-**	8.338	1.36
**DTH:F BL/6**	47.03	5.492
**DTH:F Caspase-1-/-**	5.954	0.5571
