# Supplementary material for: Metal-induced delayed type hypersensitivity responses potentiate particle induced osteolysis in a sex and age dependent manner
Source: PLoS One. 2021 May 18;16(5):e0251885. doi: 10.1371/journal.pone.0251885 (PMC8130946; doi:10.1371/journal.pone.0251885)
Supplement: S5 Table — Mean lymphocyte proliferation expression values + SEM as presented in Fig 7. (PDF) [file pone.0251885.s005.pdf]

S5 Table: Proliferation (CPM)	*Media*		*$NiCl_2$*		*$CoCl_2$*	
**Group (18-24 months old):**	**Mean**	**SEM**	**Mean**	**SEM**	**Mean**	**SEM**
**DTH:M BL/6**	894.5	94.96	958.8	103.6	861.5	174.9
**DTH:F BL/6**	1282	263.7	3957	184.8	1998	221.3
