# Supplementary material for: Metal-induced delayed type hypersensitivity responses potentiate particle induced osteolysis in a sex and age dependent manner
Source: PLoS One. 2021 May 18;16(5):e0251885. doi: 10.1371/journal.pone.0251885 (PMC8130946; doi:10.1371/journal.pone.0251885)
Supplement: S6 Table — Mean IFN-gamma production expression values + SEM as presented in Fig 8. (PDF) [file pone.0251885.s006.pdf]

S6 Table: IFN-gamma (pg / mL)	***Media***		***$NiCl_2$***		***$CoCl_2$***	
**Group (18-24 months old):**	**Mean**	**SEM**	**Mean**	**SEM**	**Mean**	**SEM**
**Vehicle:M BL/6**	15.79	1.067	19.4	1.77	16.01	3.934
**Vehicle:F BL/6**	39.2	3.584	30.56	1.107	33.25	1.087
**DTH:M BL/6**	3.481	0.9686	3.156	0.7197	3.404	0.2366
**DTH:F BL/6**	41.74	14.52	87.88	14.92	50.77	10.22
