# Supplementary material for: Metal-induced delayed type hypersensitivity responses potentiate particle induced osteolysis in a sex and age dependent manner
Source: PLoS One. 2021 May 18;16(5):e0251885. doi: 10.1371/journal.pone.0251885 (PMC8130946; doi:10.1371/journal.pone.0251885)
Supplement: S7 Table — Mean IL-17A/F production expression values + SEM as presented in Fig 8. (PDF) [file pone.0251885.s007.pdf]

S7 Table: IL-17 A/F (pg / mL)	*Media*		*$NiCl_2$*		*$CoCl_2$*	
**Group (18-24 months old):**	**Mean**	**SEM**	**Mean**	**SEM**	**Mean**	**SEM**
**Vehicle:M BL/6**	14.98	4.718	18.6	3.8	18.65	8.464
**Vehicle:F BL/6**	30.08	1.405	25.62	0.8417	18.09	2.33
**DTH:M BL/6**	14.7	2.338	11.94	1.941	15.32	0.929
**DTH:F BL/6**	22.11	5.05	39.2	8.697	30.31	5.359
