# Supplementary material for: Metal-induced delayed type hypersensitivity responses potentiate particle induced osteolysis in a sex and age dependent manner
Source: PLoS One. 2021 May 18;16(5):e0251885. doi: 10.1371/journal.pone.0251885 (PMC8130946; doi:10.1371/journal.pone.0251885)
Supplement: S8 Table — Mean osteolysis percentage expression values + SEM as presented in Fig 9. (PDF) [file pone.0251885.s008.pdf]

Table 8: P.I.O.	***% Osteolysis***	
**Group:**	**Mean**	**SEM**
**DTH:M BL/6 (18-24 months)**	8.714	0.5791
**DTH:F BL/6 (18-24 months)**	6.926	0.7156
